# What I talk about when I talk about patient self-inflicted lung injury: an ultrastructural perspective

**DOI:** 10.1186/s40635-026-00897-2

**Published:** 2026-04-13

**Authors:** Pablo Cruces, Felipe M. Llancalahuen, Carlos González, Juan P. Cruces

**Affiliations:** 1 Laboratory of Translational Research in Critical Care, Center for Research On Pandemic Resilience, Faculty of Life Sciences, Universidad Andres Bello, Santiago, Chile; 2 Unidad de Paciente Crítico Pediátrico, Hospital El Carmen Dr. Luis Valentín Ferrada, Santiago, Chile; 3 Laboratory of Translational Research in Critical Care (LTRCC), Departamento de Medicina Intensiva, Facultad de Medicina, Pontificia Universidad Católica de Chile, Santiago, Chile; 4 School of Veterinary Medicine, One Health Institute, Faculty of Life Sciences, Universidad Andres Bello, Santiago, Chile

**Keywords:** Acute lung injury, Spontaneous breathing, Respiratory effort, Patient self-inflicted lung injury

## Abstract

**Background:**

In preclinical studies, under-assisted respiratory effort has been identified as promoting lung injury, leading to the concept of “*patient self-inflicted lung injury”* (P-SILI). We aim to characterize this second hit at the ultrastructural level through scanning electron microscopy.

**Methods:**

In rats, lung injury was induced through surfactant depletion, followed by 3h of standard oxygen therapy or protective mechanical ventilation (MV). The lungs were fixed and removed to be subsequently scanned using a field emission scanning electron microscope. Images were analyzed using a semi-quantitative approach, scoring ten random alveoli for wall discontinuities and hemorrhage, and five small pulmonary vessels for microthrombosis. Computer-assisted morphometric evaluations were performed to quantify loss of lung aeration.

**Results:**

The standard oxygen therapy group showed higher frequencies of alveolar wall discontinuities, alveolar hemorrhage, and microthrombosis, compared to the MV group (all *p* < 0.05). The former also resulted in a lower aeration assessed by the aeration/tissue ratio.

**Conclusions:**

Alveolar wall fractures suggest that unsupported spontaneous breathing induces stress failure in the lung parenchyma. The fractures were associated with hemorrhage and alveolar collapse, as well as with the formation of microthrombi in small pulmonary vessels. This can be caused by regional deformation phenomena as well as by cyclical lung vascular on–off flow.

## Introduction

Under-assisted respiratory effort has been proposed as a second hit on severely and acutely injured lungs, introducing the concept of “*patient self-inflicted lung injury”* (P-SILI), a recently characterized form of lung injury with a mechanical trigger [1]. This injury is consistently characterized by lung edema and collapse, hyperemia, and alveolar hemorrhage on conventional microscopy, all of which are attenuated by invasive (controlled mechanical ventilation, MV) and non-invasive (CPAP) respiratory support therapies that override or decrease respiratory effort [2–6]. However, conventional histology lacks the resolution required to visualize ultrastructural alterations in the blood–gas barrier and microvascular phenomena, which may be critical to a deep understanding of the specific tissue targets of this mechanical injury. We aim to analyze scanning electron microscopy images of rat lungs subjected to unsupported spontaneous breathing for 3 h and to compare them with those from animals supported with controlled MV.

## Methods

This study represents a secondary analysis of an experimental study reported by our group [7]. The primary study focused on the characterization of lung and respiratory muscle injury triggered by respiratory effort, assessed by conventional microscopy. The protocol was approved by the Bioethics Committee of the Pontificia Universidad Católica de Chile (Approval Act ID 230411006, November 2024).

Acute lung injury was induced by surfactant depletion and lung collapse. Briefly, 5 ml·kg^−1^ of warm normal saline was flushed into the trachea, followed by suctioning of the residual fluid from the airway. After saline lavage, a group of 10 anesthetized rats that breathed spontaneously was maintained with standard low-flow oxygen therapy (FIO_2_ = 0.6) through a Low-Profile Nasal Anesthesia Mask (VetFloTM, Kent Scientific, USA). They were compared with a group of 10 injured rats supported with controlled MV. Animals were supported in volume-controlled ventilation with the following settings: Vt of 6 ml kg^−1^, positive end-expiratory pressure (PEEP) of 5 cmH_2_O, I:E ratio of 1:2, respiratory rate of 90/min^−1^, and a FIO_2_ of 0.6 (VentElite® Small Animal Ventilator, Harvard Apparatus, MA, USA). The subjects were observed for 3 h after stabilization. Ketamine (15 mg·kg^−1^) and xylazine (2.5 mg·kg^−1^) were administered every hour.

We performed arterial blood gases, esophageal manometry, surface electromyography on the abdominal wall, and lung transthoracic ultrasound (US) at the beginning (T0) and at the end of the observation period (T3).

Regarding gas exchange, blood samples of 100 μL from the second third of the lower tail artery were collected, using a 27G 13 mm needle (Nipro, Bridgewater, USA) connected to a 1 mL arterial blood collection BD A-LineTM syringe. Samples were assessed by a point-of-care blood analyzer (BGEM Test Cards, Epocal Inc., Siemens Healthcare Manufacturing Ltd, Dublin, Ireland).

Esophageal manometry: inspiratory effort was assessed as negative esophageal pressure swings (ΔPes). The maximal negative deflection of the esophageal pressure was defined as ΔPes, calculated over a mean of 30 s [7]. In the absence of respiratory effort, we recorded a ΔPes = 0. Afterward, the pressure rate product per minute (PRP = ΔPes x RR) was evaluated as a surrogate of work of breathing (WOB).

Surface electromyography (sEMG): it was performed on the abdominal wall to quantify expiratory effort. EMG signals were acquired by placing electrodes 2 cm apart, using an eight-channel bioamplifier, and digitized. These signals were recorded at 2000 Hz by a data acquisition software (Trigno® Avanti System; Delsys, Greater Manchester, UK). For spectral characterization, the first 5 min of filtered data were analyzed [7]. Power spectral density was computed via Welch’s method, expressed as mean frequency in Hertz (Hz).

Lung transthoracic ultrasound: the Lung US score quantified the loss of lung aeration in real time. Images were obtained using B-mode. The depth was set to 3cm, and the broadband linear array probe was set at 19 MHz (Sonosite PX Stand Series, Fujifilm Sonosite Inc., Seattle, USA). Each lung was divided into four regions: anterior right, lateral right, anterior left, and lateral left. For each zone, a score of 0–3 was assigned according to the observed pattern. The four US patterns and the scores given for each were as follows: 1) normal pattern, presence of lung sliding and artifactual horizontal A-lines (0 points); 2) B-pattern, presence of two or more well-defined vertical B-lines extending from the pleural line (1 point); 3) severe B-pattern, multiple confluent vertical B-lines extending from the pleural line (2 points); and 4) lung consolidation, presence of tissue structure with or without hyperechoic punctiform images resembling air bronchograms (3 points) [7, 8]. The lung US score was assessed by adding the worst finding in each region of interest, ranging from 0 to 12 points. The mean of two valid measurements was recorded, defined as those with a < 10% difference.

At the end of the observation period, animals were euthanized by exsanguination under deep anesthesia to facilitate the interpretation of alveolar hemorrhage. Blood samples were collected in a BD Vacutainer^®^ tube with lithium heparin, and the samples were centrifuged at 3000 rpm for 5 min to obtain plasma. Multiplex analysis for plasmatic quantification of inflammatory (interleukin-1β and tumor necrosis factor-α) and immunomodulatory (Interleukin-2) cytokines, chemokine (growth-regulated protein α, GRO-α), and soluble endothelial adhesion molecules (intercellular adhesion molecule-1 and vascular cell-adhesion molecule-1) was performed using Luminex 200^®^ (Luminex Corporation, Austin, USA).

After confirming the absence of vital signs, a bolus of 4% formaldehyde and 0.5% glutaraldehyde in 0.2 M HEPES buffer (pH 7.4) was introduced in a liquid column into the airway until a pressure of 25 cmH_2_O was reached, fixing the lung tissue. Subsequently, the chest cavity was opened, and the lungs were removed, and their basal regions were examined. Four lungs from the standard oxygen therapy group and five from the MV group were randomly selected and examined using a field emission scanning electron microscope (LEO 1450VP). For scanning, lungs were processed for critical point drying and coated with gold.

Field emission scanning electron microscopy micrographs were analyzed using a semi-quantitative approach at the animal level. For each subject, ten alveoli were randomly selected from non-overlapping fields with adequate tissue preservation and clear visualization of alveolar septa. Each alveolus was scored as positive [1] or negative (0) for (i) alveolar wall discontinuity (visible break in septal continuity consistent with a microfracture) and (ii) alveolar hemorrhage (erythrocytes within the alveolar airspace). In parallel, five small pulmonary vessels per subject were randomly selected from non-overlapping fields and scored as positive [1] or negative (0) for microthrombosis, defined as an intraluminal aggregate compatible with thrombus formation partially or completely occupying the vessel lumen. For each endpoint, results were expressed as positive structures per fixed denominator (0–10 for alveoli; 0–5 for microthrombosis), equivalent to the proportion of examined structures exhibiting the alteration. Image scoring was performed on coded images by an investigator blinded to group allocation (CG).

Next, computer-assisted morphometric evaluations were performed to determine lung aeration, assessed as area (μm^2^) and aeration/tissue ratio (ImageProPlus®, ver. 7.0.1.658, Media Cybernetics, Rockville, MD, USA) [6].

### Statistical analysis

Normality of the data was evaluated using the Shapiro–Wilk test. Welch’s t-test was applied when all groups satisfied normality; otherwise, the Mann–Whitney U test was used. Data were presented as median (IQR). Significance was set at *p* < 0.05. All statistical analyses were performed in Python using SciPy v1.11.1.

## Results

There were no differences in weight, sex, and RR between the standard oxygen therapy and MV groups. During the experimental protocol, mortality occurred in three subjects in the former, all of them in the last hour of observation, with no mortality in the latter. Lungs from these subjects were also considered for morphological assessments.

PaO_2_ showed no differences between groups during the overall experimental period, but the standard oxygen therapy group tended to have higher PaCO_2_ than the MV group at T0 and T3. Standard oxygen therapy resulted in higher ΔPes, PRP, and sEMG expiratory effort than the MV group throughout the study, and higher lung US score at T0 (all *p* < 0.01) (Table 1). The standard oxygen therapy group also resulted in higher plasma chemokine GRO-α levels than the MV group (13.2 [11.0] vs 58.5 [65.8] pg/mL, *p* < 0.05). There were no other differences in any other biomarker between groups.

**Table 1:** Physiological data for the experimental groups at the beginning (T0) and end of the observation period (T3)

Variables	Standard oxygen theraphy	Mechanical ventilation	P-value
Weight (g)	320 (17.5)	322 (30)	0.86
Sex (male/female)	5/5	5/5	1.0
RR T0 (breaths/min)	103.0 (30.0)	90.0 (0.0)	0.62
RR T3 (breaths/min)	82.0 (20.5)	90.0 (0.0)	0.11
PaO_2_ T0 (mmHg)	84.8 (26.7)	94.7 (134.7)	0.27
PaO_2_ T3 (mmHg)	91.5 (31.8)	171.6 (159.0)	0.31
PaCO_2_ T0 (mmHg)	66.6 (21.7)	48.1 (22.0)	0.09
PaCO_2_ T3 (mmHg)	61.2 (15.2)	47.2 (13.0)	0.13
ΔPes T0 (cmH_2_O)	0.8 (0.2)	0.0 (0.0)	< 0.01
ΔPes T3 (cmH_2_O)	1.1 (0.2)	0.0 (0.0)	< 0.01
PRP T0 (cmH_2_O/min)	50.9 (8.2)	0.0 (0.0)	< 0.01
PRP T3 (cmH_2_O/min)	74.4 (49.7)	0.0 (0.0)	< 0.01
sEMG T0 (Hz)	97.5 (41.2)	45.7 (24.0)	< 0.01
sEMG T3 (Hz)	93.8 (30.1)	54.4 (27.0)	< 0.01
Lung US score T0	6.0 (1.0)	3.0 (1.8)	< 0.01
Lung US score T3	6.0 (3.0)	3.0 (2.8)	0.09

Data are expressed as median (interquartile range)

Significant within-group differences are denoted by *p* < 0.05

RR, respiratory rate; PaO_2_, partial pressure of oxygen in arterial blood; PaCO_2_, partial pressure of carbon dioxide in arterial blood; ΔPes, swing of esophageal pressure; PRP, pressure rate product; sEMG, surface electromyography on the abdominal wall; US, ultrasound

**Fig. 1:**
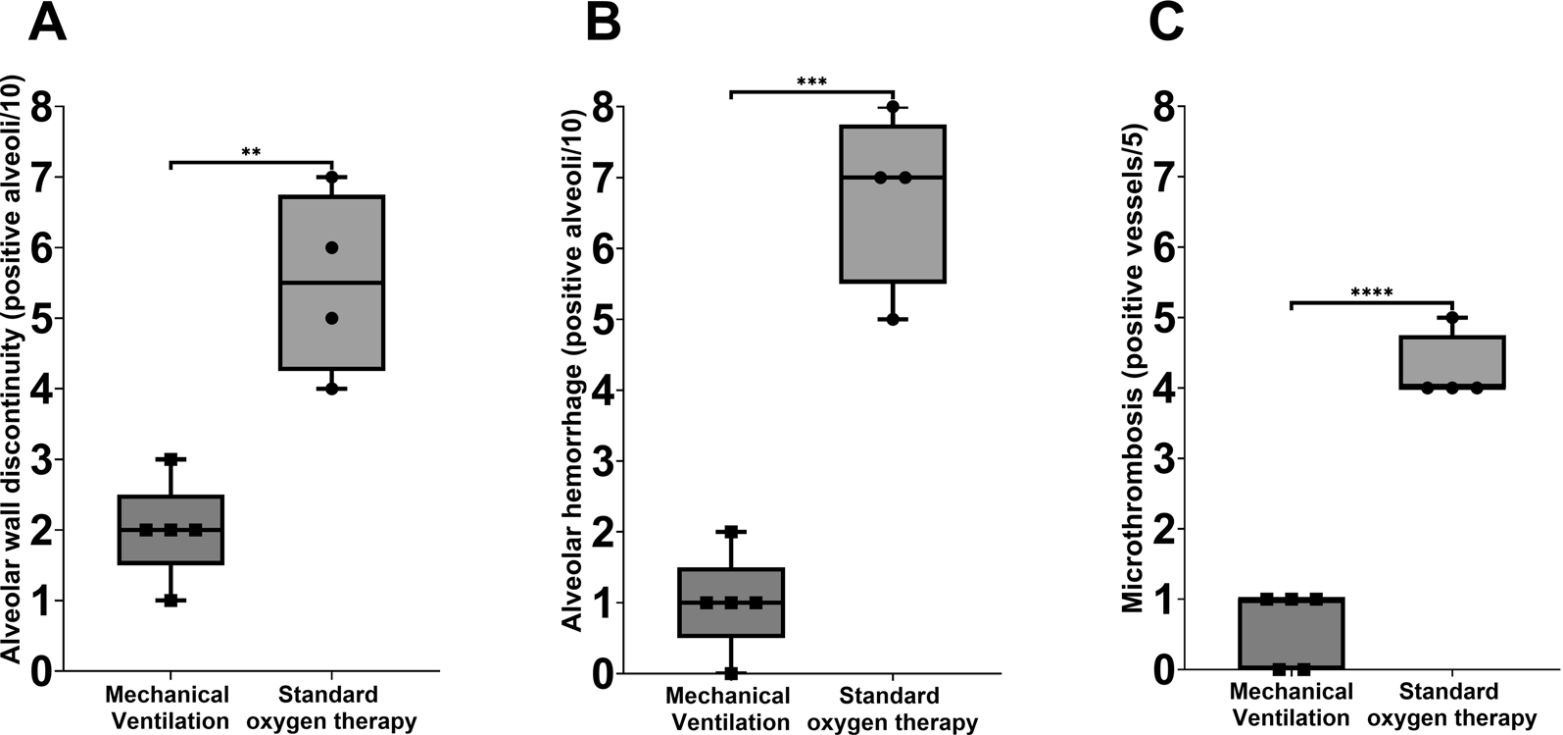
Semi-quantitative field emission scanning electron microscopy scoring of ultrastructural alterations (box-and-whisker plots with individual animals). Semi-quantitative scoring was performed at the animal level by randomly sampling 10 alveoli per subject to assess alveolar wall discontinuity (**A**) and alveolar hemorrhage (**B**), and 5 small pulmonary vessels per subject to assess microthrombosis (**C**). Outcomes are displayed as the number of positive structures per fixed denominator (alveoli: n/10; vessels: n/5). Each symbol represents one animal (Standard oxygen therapy, *n* = 4; Mechanical Ventilation, *n* = 5). Box-and-whisker plots show the median (center line) and interquartile range (box), with whiskers indicating the minimum to maximum values. Groups were compared using a two-tailed Mann–Whitney U test. Significance is indicated as *p* values or asterisks (*p* < 0.05; *p < 0.01; **p < 0.001; ***p < 0.0001)

**Fig. 2:**
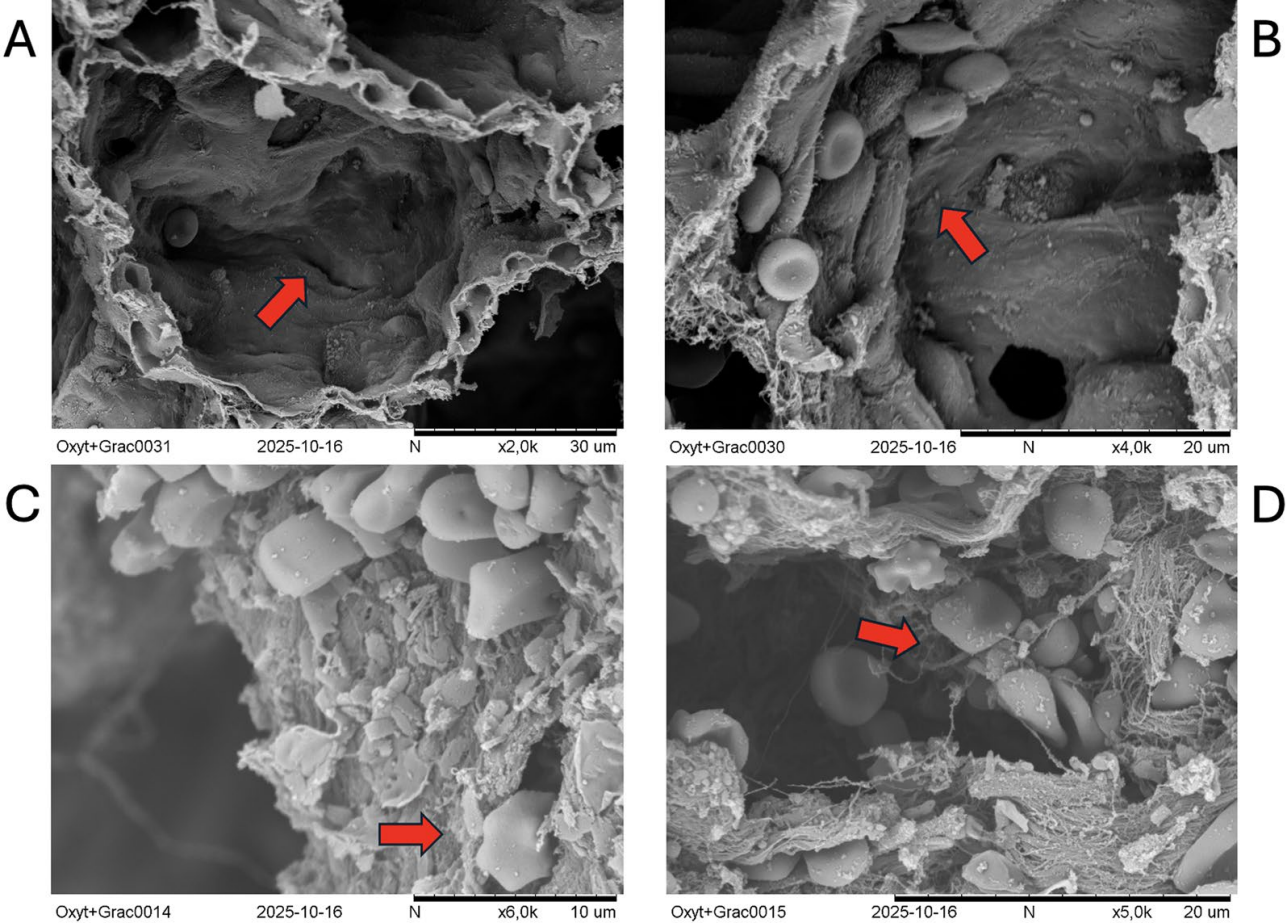
Field emission scanning electron microscopy of the injured and unsupported lungs (standard oxygen therapy), showing a severely disrupted alveolar structure, abundant, dense, and disorganized cellular and surfactant debris, fragmented septa, and an irregular and shredded surface appearance, highlighting microfractures in the alveolar walls (**A**), pulmonary hemorrhage (**B**), alveolar macrophage infiltration (**C**), and microthrombosis in small vessels (**D**)

**Fig. 3:**
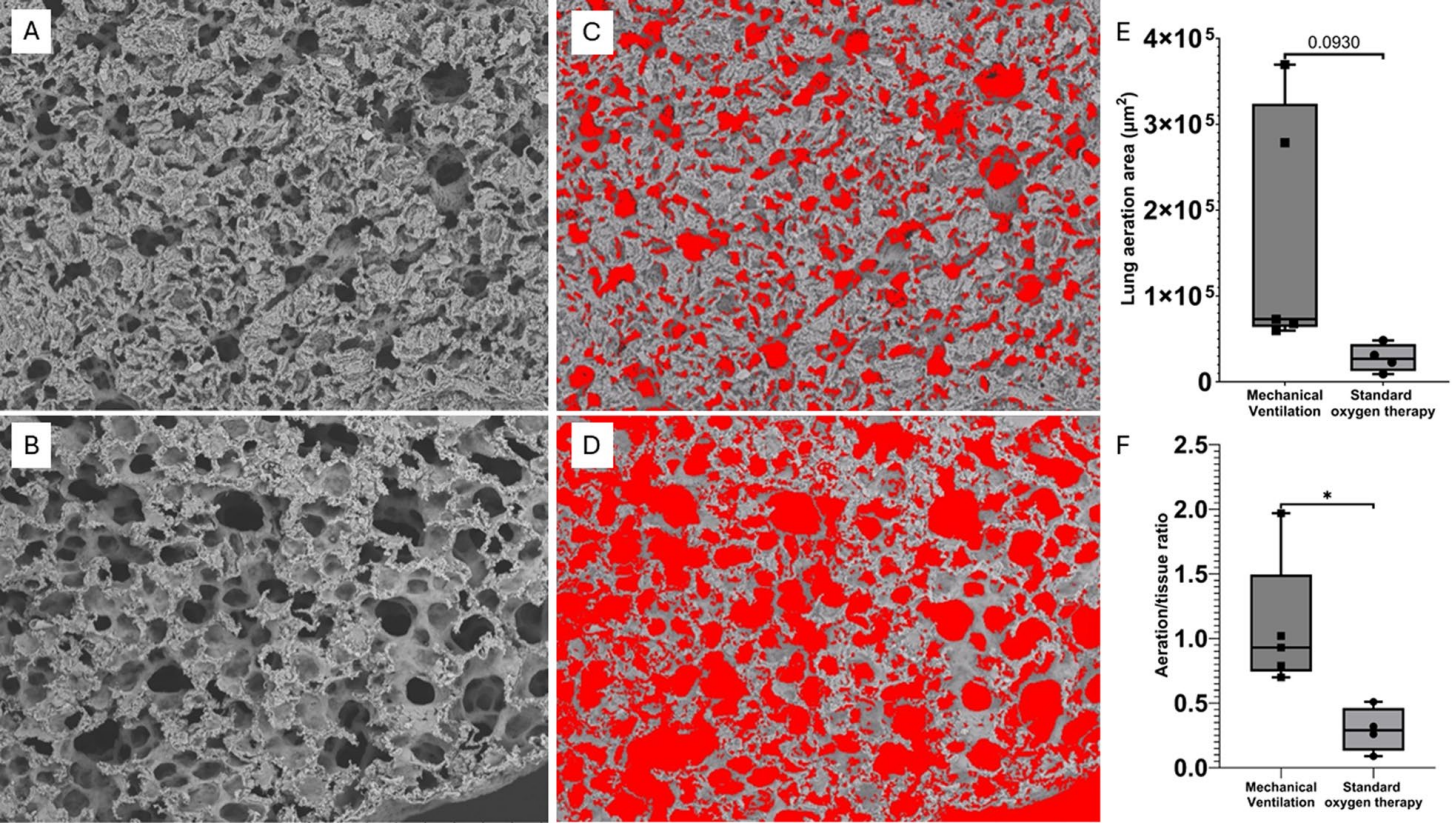
Morphometric analysis of lung aeration derived from scanning electron microscopy. **A** and **B** show representative scanning electron microscopy images for each experimental group. Aerated alveolar areas were highlighted in red (**C and D**). Each symbol represents one animal (standard oxygen therapy, *n* = 4; mechanical ventilation, *n* = 5). Box-and-whisker plots show the median (center line) and interquartile range (box) for lung aeration metrics, with whiskers indicating the minimum to maximum values (**E and F**). Groups were compared using a two-tailed Mann–Whitney U test. Significant within-group differences are denoted by *p* < 0.05

The standard oxygen therapy group showed higher frequencies of alveolar wall discontinuities, alveolar hemorrhage, and microthrombosis compared to the MV group (all *p* < 0.05, Fig. 1). Occasionally, we also identify infiltration by alveolar macrophages only in this group, shown in Fig. 2. The standard oxygen therapy group resulted in a lower aeration/tissue ratio than the MV group (*p* < 0.05) and showed a strong trend toward a larger aeration area (Fig. 3).

**Fig. 4:**
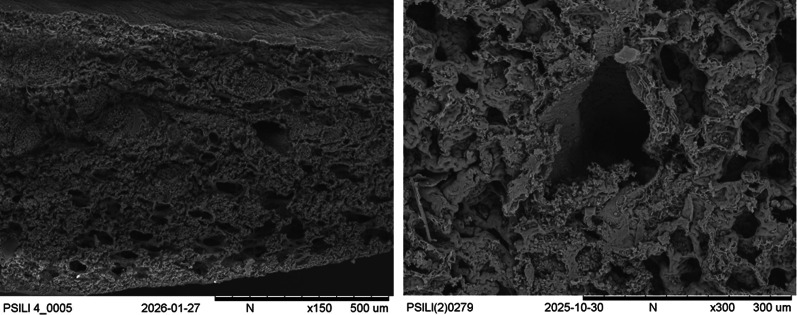
Field emission scanning electron microscopy of the injured and unsupported lungs (standard oxygen therapy), showing the usual collapse of alveolar units, coexisting in the same field with the overdistension of those that remained aerated, which generated inhomogeneity. The same phenomenon was observed in different regions, and was shown with varying magnification

Scanning electron microscopy also frequently identified alveolar collapse and heterogeneity of regional aeration in the standard oxygen therapy group, with collapsed and overinflated alveoli coexisting in the same field (representative images in Fig. 4).

## Discussion

In a three-hour severe P-SILI model, we showed the ultrastructural effects of unsupported spontaneous breathing on the lungs, highlighting the highest rate of alveolar wall fractures and their prevention through controlled MV. On the epithelial side of the blood–gas barrier, these fractures caused alveolar hemorrhage and loss of lung aeration, while on its vascular side, they are spatially related to the formation of microthrombi. Thus, these results suggest that unsupported spontaneous breathing amplifies damage to alveolar walls and microvasculature in severely injured lungs.

The ultrastructural findings extend beyond the classical histological pattern of P-SILI already reported in prior studies [3, 5]. The presence of fractures in the alveolar walls and the extent of microthrombotic phenomena had not been previously identified due to the resolution limitations of conventional microscopy [2–7]. Alveolar hemorrhage and collapse, widely reported in other P-SILI studies, are spatially related to alveolar wall disruptions, suggesting that microfractures were the primary mechanism of injury, while the alveolar flooding is secondary to the disruption of the blood-gas barrier. Accordingly, the loss of lung aeration was identified in in vivo and ex vivo assessments of the unassisted group. In turn, the endothelial injury caused by these microfractures would explain the activation of coagulation at the ultrastructural level, generating microthrombi. Concordantly, in a clinical setting, direct and indirect markers of coagulation activation, such as thrombocytopenia, elevated D-dimer levels, and alveolar dead space, are consistently associated with worse outcomes in acute respiratory distress syndrome [9–12].

Microfractures were suggestive of stress failure and probably associated with increased obligatory and accessory respiratory muscle activity compared to the MV group, generating regional tissue deformation and large cyclic blood flow oscillations [2, 4, 6, 7]. These are consistent with the greater inspiratory effort, expiratory effort, and WOB in the unassisted group, triggered by increased respiratory drive caused by respiratory acidosis, systemic inflammation, and the activation of mechanosensitive lung tissue receptors. In addition to promoting alveolar collapse, these are plausible explanations for regional heterogeneity and may contribute to pulmonary air leak. Heterogeneity and gas redistribution are known stress raisers in acutely injured lungs, generating a synergistic increase in regional transpulmonary pressures [2, 4, 13, 14]. The Macklin effect is a mechanism in which alveolar rupture is caused by large transpulmonary pressure swings; it induces air leak, a serious complication in patients breathing strongly on spontaneous ventilation [15, 16].

This study has the limitations inherent to small-animal studies and to the exploratory nature of scanning electron microscopy. These findings should be interpreted as morphological correlates rather than direct proof of mechanical failure mechanisms. However, we believe that the ultrastructural findings allow for a better understanding of the pathophysiological mechanisms associated with the *“second hit”* generated by unsupported spontaneous breathing on severely and acutely injured lungs, which directly injures the blood-gas barrier, affecting both the alveoli (hemorrhage and loss of aeration) and small pulmonary vessels (microthrombosis).

## Data Availability

The images are available from the corresponding author on reasonable request.
